# Effect of Ti Content on the Microstructure and High-Temperature Creep Property of Cast Fe-Ni-Based Alloys with High-Al Content

**DOI:** 10.3390/ma14010082

**Published:** 2020-12-26

**Authors:** Gokul Obulan Subramanian, Changheui Jang, Ji Ho Shin, Chaewon Jeong

**Affiliations:** Korea Advanced Institute of Science and Technology, Daejeon 34141, Korea; gokul@kaist.ac.kr (G.O.S.); shinjiho@kaist.ac.kr (J.H.S.); rue0507@kaist.ac.kr (C.J.)

**Keywords:** heat resistant alloys, alumina-forming alloys, γ’-Ni_3_(Al,Ti) precipitates, B2-NiAl phase, creep

## Abstract

The cast Fe-Ni-based austenitic heat-resistant alloys with 4.5 wt% Al and varying Ti content were developed for high-temperature application. With increase in Ti content, strength of model alloys increased gradually at 700 °C and 750 °C. At 750 °C, alloys with 35Ni–(2~4)Ti composition showed a significant increase in creep rupture life compared to 30Ni–1Ti alloy, attributed to the increase in γ’-Ni_3_(Al,Ti) precipitates due to higher Ni and Ti content. Among the 35Ni–(2~4)Ti alloys, increasing Ti content from 2 to 4 wt% gradually increased the creep rupture life in the as-cast condition. The creep rupture life was improved after solution annealing treatment, however, the beneficial effect of higher Ti content was not evident for 35Ni–(2~4)Ti alloys. After solution annealing, interdendritic phases were partially dissolved, but coarse B2-NiAl phases were formed. The size and amount of coarse B2-NiAl phases increased with Ti content. In the creep-tested specimens, creep void nucleation and crack propagation were observed along the coarse B2-NiAl phases, especially for high-Ti alloys. Therefore, the beneficial effect of the increase in γ’-Ni_3_(Al,Ti) precipitates for high-Ti alloys on creep property was limited due to the detrimental effect of the presence of coarse B2-NiAl phases.

## 1. Introduction

The advanced ultra-supercritical (A-USC) power plants and Generation IV (Gen IV) nuclear reactors are designed to achieve higher thermal efficiencies by utilizing higher operating temperature and pressure. To maintain the structural integrity, it is necessary for the structural materials to possess excellent mechanical strength and creep property for longer operational periods. The A-USC power plant would typically utilize the steam Rankine cycle to drive the turbines [1], while the supercritical CO_2_ (S-CO_2_) Brayton cycle is a promising candidate for Gen IV nuclear reactors [2,3]. Both the high-temperature steam and S-CO_2_ environments are aggressive toward the structural materials, which causes environmental degradation by oxidation. Ni-based alloys with sufficient Cr content show better oxidation resistance than Fe-based alloys with lower Cr content [4,5,6]. Furthermore, the Ni-based alloys exhibit superior creep life. Meanwhile, the oxidation resistance also affects the creep life of the material, such that the creep life of Ni-based Alloy 600 was significantly shortened in the aggressive S-CO_2_ environment with higher oxidation rates [7]. Therefore, it is important to use structural materials with optimal chemical composition that can provide a balanced creep property and oxidation resistance.

The Fe-Ni-based alumina-forming alloys with a compositional range of (20~32)Ni–(2~4)Al (wt%) provide the formation of a protective alumina (Al_2_O_3_) layer in the oxidizing environment and show good creep property from γ’-Ni_3_Al precipitates, Laves (Fe_2_Nb) phases, carbides, and B2-NiAl phases [8,9,10,11,12,13,14,15,16,17,18,19]. It is typically preferable to have higher matrix Al content for the application in the highly oxidizing environments, since it was reported that the gradual depletion of Al in the subsurface for the 3.5 wt% Al alloy resulted in breakaway oxidation during prolonged exposure in the S-CO_2_ environment [20]. Furthermore, the addition of Ti increases the amount of γ’-Ni_3_(Al,Ti) precipitates and creep life, but deteriorates the oxidation resistance of the alumina-forming alloys [11,21]. Therefore, it is challenging to develop cost-effective alloys that are oxidation-resistant with improved creep property in high-temperature aggressive environments. In this regard, we have explored the possibilities of Fe-Ni-based heat-resistant alloys with increased Al content for high-temperature oxidation resistance, and higher Ti content for increased γ’-Ni_3_(Al,Ti) precipitates to improve the creep life. Accordingly, the Fe-Ni-based heat-resistant alloys with 4.5 wt% Al and 1–4 wt% Ti content were developed and the creep rupture life with microstructural evolution were evaluated at 750 °C.

## 2. Materials and Methods

### 2.1. Materials

Austenitic Fe-Ni-based heat resistant alloys with a nominal composition of Fe–(30~35)Ni–(16~18)Cr–4.5Al–(1~4)Ti–1Nb–C (in wt%) have been considered in this study. The higher Al content (4.5 wt%) was selected to facilitate sufficient Al supply to the exposed surface in highly oxidizing environments. Ni content was kept at 30–35 wt% and Cr content at 16–18 wt% to maintain the austenite matrix. The Ti content was controlled in the range of 1–4 wt% for increased γ’-Ni_3_(Al,Ti) precipitates. In addition, 1 wt% Nb was used for additional strengthening with Laves phases and NbC carbides.

A series of model alloys with different compositions were prepared as shown in Table 1. The alloys were named with the numbers corresponding to the nominal Al and Ti content; for instance, alloy 43 refers to the 4.5 wt% Al and 3 wt% Ti present in the alloy. Minor elements Zr and B were added in some of the alloys, which are known to segregate towards the grain boundaries and enhance the creep and oxidation properties [11,22]. In addition, increasing the C content in cast alumina-forming alloys was reported to substantially enhance the creep rupture life [23]. To indicate the presence of beneficial minor elements, the suffix ‘Z’ was included to denote only Zr addition and ‘+’ to denote Zr and B addition with increased C content. The Ni content in alloy 43 was increased to 35 wt% from the 30 wt% in 41Z to compensate for the higher Ti content in alloy 43. The alloys 41Z and 43 were intended to check the effect of increased Ni and Ti content on the high-temperature mechanical properties. Furthermore, the alloys 42+ to 44+ were prepared to evaluate the effect of higher Ti content (2 to 4 wt%) with creep life enhancing minor elements.

### 2.2. Experimental Methods

All the model alloys provided in Table 1 were prepared using vacuum arc remelting technique in an argon atmosphere to make ingots of 250 g to 1.2 kg, denoted as as-cast (AC) condition. A small portion of the ingot was cut and solution annealed at 1200 °C in air for 15 min followed by water quenching, denoted as solution annealed (SA) condition. Tensile and creep specimens were fabricated from AC condition, while only creep specimen was fabricated from SA condition due to a limited amount of materials. The tensile tests were conducted at 700 and 750 °C in air at a displacement rate of 0.1 mm/min using mini-sized tensile specimens with gauge length of 5 mm and thickness of 0.5 mm. The tensile test was performed 30 min after reaching the test temperature. Air creep tests were conducted at 750 °C and 150 MPa using plate-type specimen with gauge length of 16 mm and thickness of 1.5 mm using a dead-weight type machine. Due to the limited availability of tensile and creep specimens, the tests were conducted once for each material and test condition. 

For microstructural analysis, the specimens were polished to 1 μm diamond paste and observed using a field emission scanning electron microscope (SEM, SU5000, Hitachi, Tokyo, Japan) equipped with an energy dispersive X-ray spectroscope (EDS, Oxford instrument, Abingdon, Oxfordshire, UK) in the backscattered electrons (BSE) mode. Electron backscatter diffraction (EBSD, Quattro S, FEI Co., Hillsboro, OR, USA) and X-ray diffraction (XRD, Rigaku D/MAX-2500, Rigaku Co., Tokyo, Japan) analysis were used to characterize the overall phase content of the specimens. The transmission electron microscopy (TEM) specimens were taken from the crept specimens within 3 mm from the fractured surface using a focused ion beam system (FIB, Helios Nanolab 450 F1, FEI Co., Hillsboro, OR, USA). The nano-scale microstructure was characterized using TEM (Talos F200X, FEI Co., Hillsboro, OR, USA) equipped with an energy dispersive spectrometer (STEM-EDS, Super-X EDX detector, FEI Co., Hillsboro, OR, USA) at 200 kV. The crystal structures of the nano-scale phases were identified either by the selective area diffraction (SAD) pattern or high-resolution transmission electron microscopy (HRTEM) mode coupled with the fast Fourier transform (FFT) processing technique.

## 3. Results

### 3.1. Phase Stability Calculation

**Figure 1:**
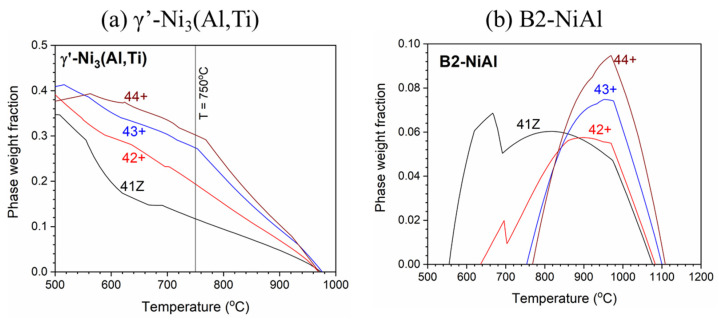
Thermodynamic phase stability of (**a**) γ’-Ni_3_(Al,Ti) and (**b**) B2-NiAl phase for the model alloys.

The thermodynamic phase stability calculations were performed for the model alloys using Thermo-Calc program with TCFE9 database. The thermodynamic phase stability of the γ’-Ni_3_(Al,Ti) and B2-NiAl phases for the model alloys are shown in Figure 1. The fraction of γ’-Ni_3_(Al,Ti) precipitates gradually increased from the alloy 41Z to 44+ at 750 °C. Meanwhile, the formation temperature of B2-NiAl phases was around 560 °C for alloy 41Z and pushed towards 640 °C for alloy 42+. Further increasing the Ti content to 3 and 4 wt% (alloys 43+ and 44+) moved the formation temperature over 750 °C. Additionally, the B2-NiAl phase fraction and solvus temperature increased with increasing Ti for alloys 42+ to 44+. The B2-NiAl phase fraction for alloys 42+ to 44+ reached maximum around 900–970 °C, while the solvus temperature is in the range of 1080–1110 °C. According to Figure 1, the γ’-Ni_3_(Al,Ti) precipitates would be predominant over B2-NiAl phases for the high-Ti alloys (43+ and 44+) at 750 °C where creep tests were performed.

### 3.2. Microstructure of Cast Alloys

**Figure 2:**
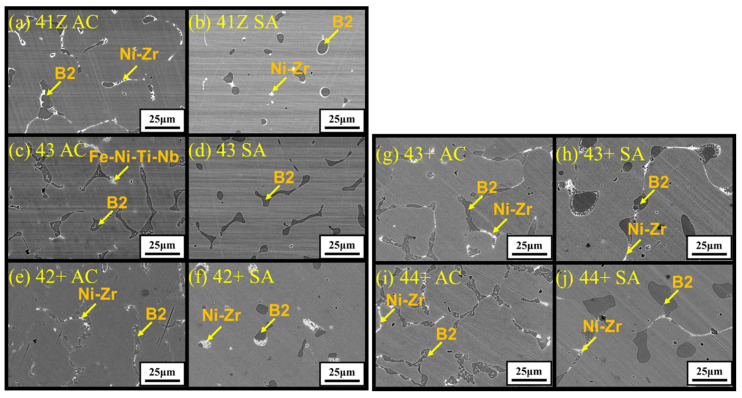
SEM-BSE images showing the microstructure of the developed alloys (**a**) 41Z AC, (**b**) 41Z SA, (**c**) 43 AC, (**d**) 43 SA, (**e**) 42+ AC, (**f**) 42+ SA, (**g**) 43+ AC, (**h**) 43+ SA, (**i**) 44+ AC, and (**j**) 44+ SA. ID B2 = Interdendritic B2-NiAl phase.

**Figure 3:**
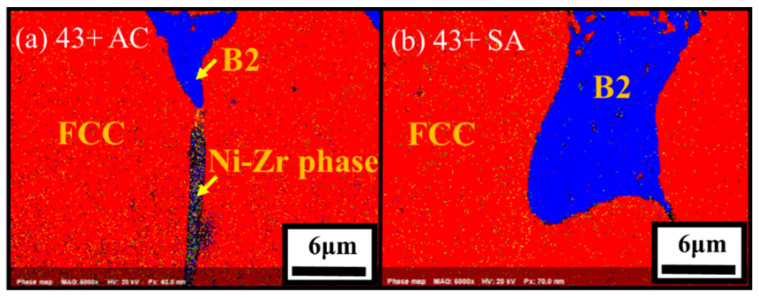
EBSD phase mapping for the alloys (**a**) 43+ AC and (**b**) 43+ SA.

The microstructure of the model alloys in as-cast and solution annealed conditions are shown in Figure 2. All the alloys showed dendritic microstructure with austenite matrix and interdendritic phases in as-cast condition. From the SEM-EDS analysis in Table 2, the dark interdendritic phases were identified as Ni-Al rich phase. The bright interdendritic phase correspond to Fe-Ni-Ti-Nb rich phase for alloy 43 and Ni-Zr rich phase for other alloys. The EBSD phase mapping of alloy 43+ in Figure 3 showed the dark Ni-Al rich phase having B2-NiAl structure in both as-cast and solution annealed conditions, while the structure of the bright Ni-Zr phase could not be clearly identified. Meanwhile, the Ni-Zr phase was previously reported as a Ni_7_Zr_2_ intermetallic phase in Zr-containing Fe-based alloys [24,25]. Similarly, the observed Ni-Zr phase in the model alloys would be Ni_7_Zr_2_, but the detailed TEM analysis would be needed for clarification. The solution annealing partially dissolved the interdendritic B2-NiAl and Ni-Zr rich phases as observed in Figure 2, whereas the Fe-Ni-Ti-Nb-rich phases in alloy 43 were completely dissolved. For alloy 43, the remaining B2-NiAl phases after solution annealing exhibited dendritic morphology similar to the as-cast condition, while it exhibited coarse blocky morphology for other alloys. The B2-NiAl interdendritic phases in the as-cast condition showed a width of approximately 3–4 μm and length of 20–30 μm. Meanwhile, solution annealing coarsened the B2-NiAl phases, especially for the alloys 43+ and 44+. After solution annealing, the average width of coarse B2-NiAl phases were measured and it increased with Ti: 3.8 μm (41Z SA), 4.7 μm (42+ SA), 7.7 μm (43+ SA), and 8.4 μm (44+ SA).

**Figure 4:**
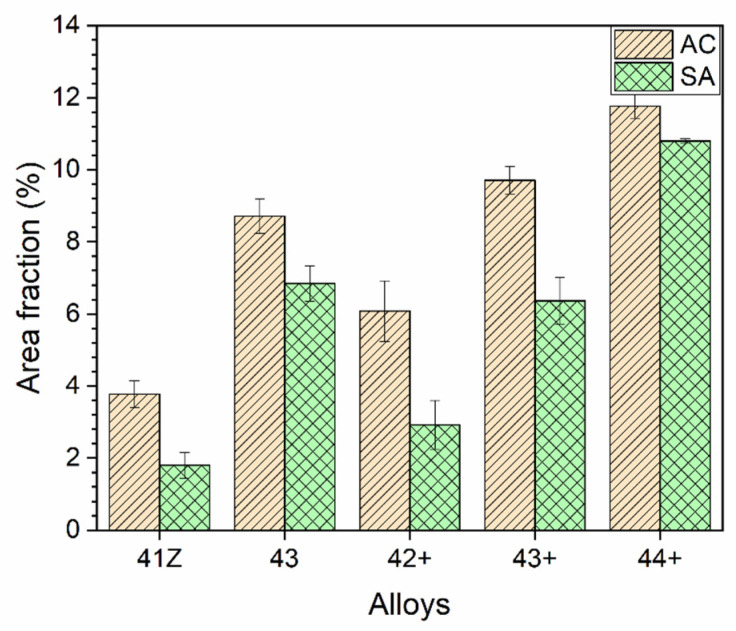
Area fraction of the B2-NiAl phases for the developed alloys in as-cast and solution annealed conditions.

Meanwhile, the area fraction of the B2-NiAl phases was measured and the results are shown in Figure 4. The area fraction of B2-NiAl phases increased with Ti in as-cast conditions, up to 12%. For all alloys, the area fraction of B2-NiAl phases was reduced after solution annealing, suggesting partial dissolution. Meanwhile, the reduction of the interdendritic B2-NiAl phases was less for alloy 44+ (with the highest Ti) compared to alloys with lower Ti. On the other hand, the area fraction of Ni-Zr rich phases were 1–2% for the alloys in the as-cast condition, which were further reduced to 0.5–1% after solution annealing.

**Figure 5:**
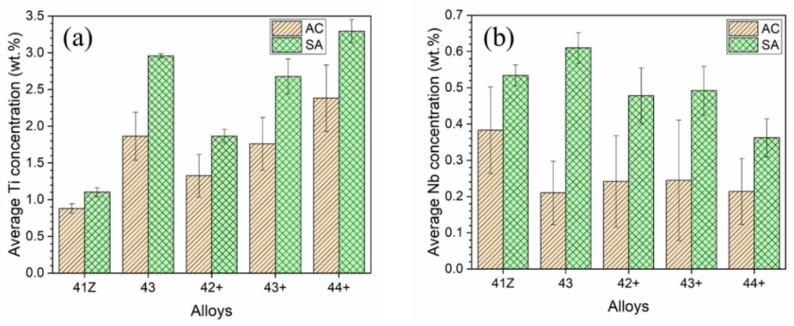
Average concentration of (**a**) Ti and (**b**) Nb in the austenite matrix in as-cast (AC) and solution annealed (SA) conditions, from SEM-EDS analysis.

The SEM-EDS analysis results for the interdendritic phases in alloy 43+ are summarized in Table 2. As shown in Table 2, for the B2-NiAl phases, Ti content was substantially reduced after solution annealing, while Al did not change noticeably. In the case of Ni-Zr phases, considerable reduction in Si content was observed after solution annealing. Meanwhile, the partial dissolution of the interdendritic B2-NiAl and Ni-Zr phases after solution annealing resulted in increase in Ti and Nb contents in the austenite matrix, as shown in Figure 5.

### 3.3. High-Temperature Mechanical Properties

**Figure 6:**
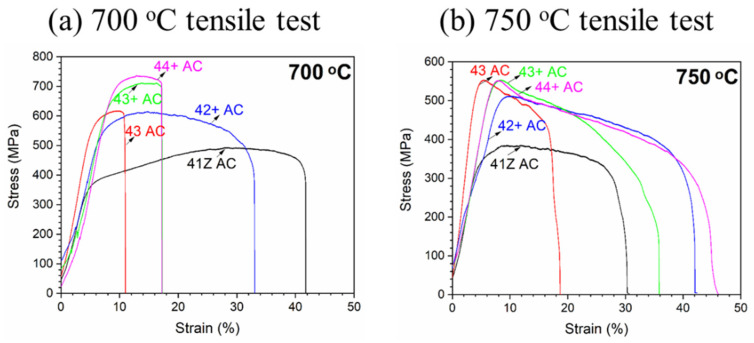
Tensile test results of the model alloys in as-cast condition at (**a**) 700 °C and (**b**) 750 °C.

Figure 6a,b shows the stress-strain curves of tensile tests of the model alloys in as-cast condition at 700 and 750 °C, respectively, and strength and elongation are tabulated in Table 3. Strain hardening behavior was observed at 700 °C for the alloys, but it was negligible at 750 °C due to the occurrence of dynamic recovery. The alloy 43 AC showed a considerable increase in tensile strength from alloy 41Z AC at 700 and 750 °C because of the increased Ni and Ti content leading to a higher fraction of γ’-Ni_3_(Al,Ti) precipitates (Figure 1). The tensile strength also increased from alloy 42+ AC to 43+ AC at both temperatures, in which the increased Ti content also increases the fraction of γ’-Ni_3_(Al,Ti) precipitates (Figure 1). Nevertheless, a moderate increase in tensile strength was observed from alloy 43+ AC to 44+ AC at 700 °C, while it was comparable at 750 °C. Meanwhile, the tensile strength of the alloy 43+ AC was higher than alloy 43 AC at 700 °C, which could be attributed to the presence of Zr and B along with higher C content. However, the effect of those minor elements on strengthening was not observed at 750 °C in which alloy 43 AC and 43+ AC showed comparable strength. At 700 °C, the elongation of high-Ti alloys (43, 43+ and 44+) were lower than the alloys 41Z and 42+ in as-cast condition. The lower elongation of the high-Ti alloys can be associated with the presence of compositional inhomogeneity and higher fraction of interdendritic phases that considerably limits plastic deformation by strain hardening. At 750 °C, the elongation was somewhat increased for the high-Ti alloys at the expense of strength (Table 3).

**Figure 7:**
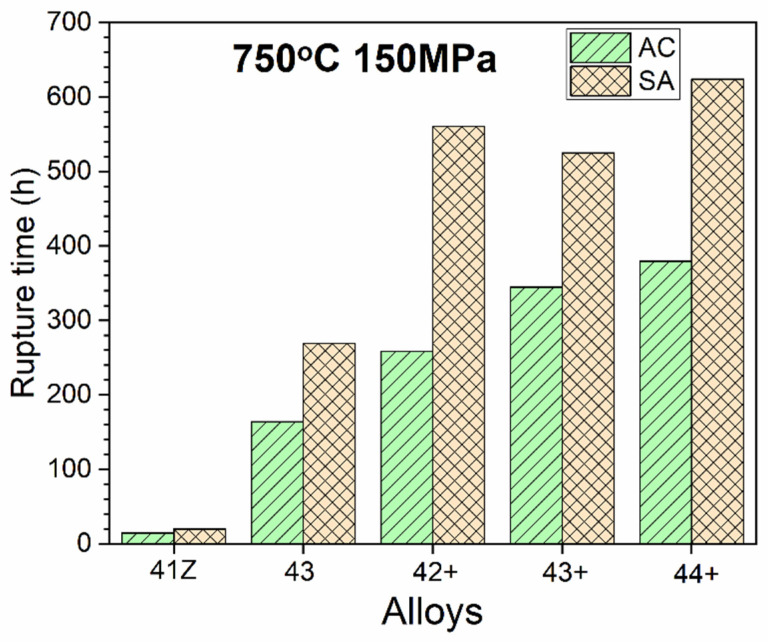
Creep rupture life of the alloys in air at 750 °C and 150 MPa.

The creep rupture life of the alloys tested at 750 °C and 150 MPa in air are shown in Figure 7. The alloy 41Z exhibited the least creep rupture life among the model alloys despite the presence of Zr. Meanwhile, the creep rupture life improved significantly for alloy 43, indicating the beneficial effect of increasing Ni and Ti contents. It can be also observed that the alloy 43+ with the beneficial minor elements and increased C content showed higher creep rupture life than alloy 43, emphasizing the importance of minor elements for enhanced creep rupture life. Meanwhile, the creep rupture life for alloys 42+ to 44+ gradually increased with Ti in the as-cast condition. The solution annealing resulted in higher creep rupture life for all model alloys, which may be related to the reduction of interdendritic phases, and increase in Ti and Nb contents of the austenite matrix as described in the previous section. However, in the solution annealed condition, there is no clear effect of Ti content on creep rupture life as the alloys 42+ to 44+ showed similar creep rupture life. To understand such behavior, the observed creep rupture life will be discussed in view of the microstructure evolved during the creep test in the next section.

### 3.4. Microstructure of Crept Alloys

**Figure 8:**
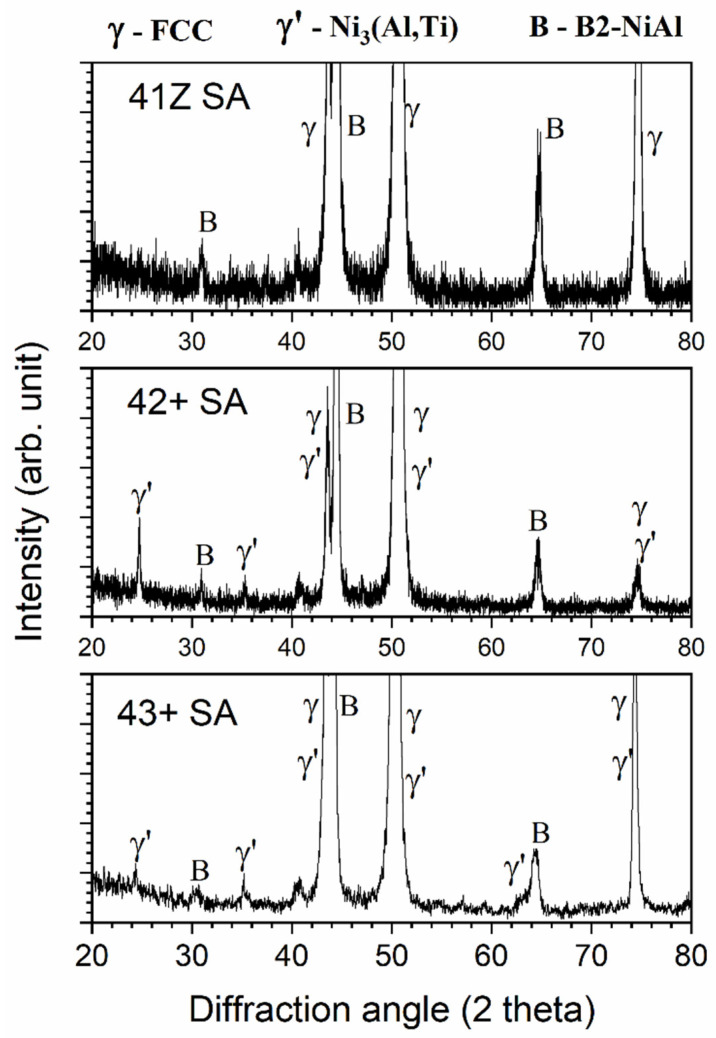
XRD results for the alloys 41Z SA, 42+ SA, and 43+ SA after the creep test in air at 750 °C and 150 MPa.

XRD analysis was conducted on the crept specimen of alloys 41Z SA, 42+ SA, and 43+ SA, and the results are shown in Figure 8. All the crept specimens exhibited peaks of B2-NiAl phase as well as those of the austenite matrix. Meanwhile, distinct peaks of γ’-Ni_3_(Al,Ti) precipitates can be identified for alloys 42+ SA and 43+ SA with increased Ni and Ti content, while they were absent for the alloy 41Z SA.

**Figure 9:**
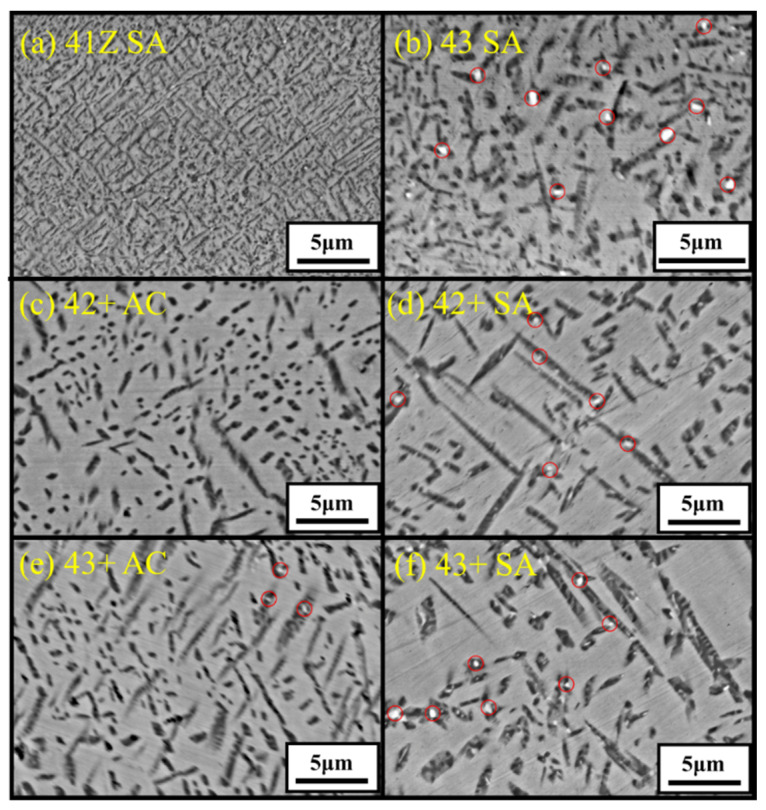
SEM-BSE images showing the microstructure near (3 mm away from) the fracture region for alloys (**a**) 41Z SA, (**b**) 43 SA, (**c**) 42+ AC, (**d**) 42+ SA, (**e**) 43+ AC, and (**f**) 43+ SA after the creep test in air at 750 °C and 150 MPa. Laves phases are encircled.

**Figure 10:**
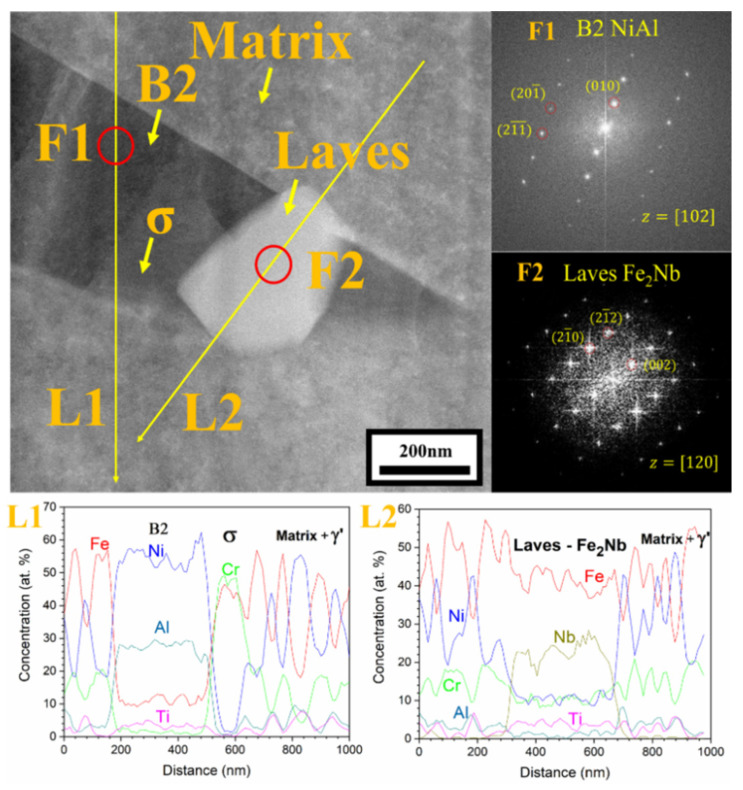
STEM-EDS line scan analysis with FFT pattern for the needle-like B2-NiAl phases and adjacent Laves phase in alloy 43+ SA after the creep test in air at 750 °C and 150 MPa.

Detailed microstructural analysis using SEM were conducted for the area within 3 mm from the fractured region of the crept specimens and the results are shown in Figure 9. The microstructural analysis near the fractured region shows extensively precipitated phases in the austenite matrix compared to the plain austenite matrix before the creep test (Figure 2). The dark and needle-like phase was enriched in Ni and Al based on the STEM-EDS line scan analysis L1 in Figure 10, and identified as B2-NiAl phase from the HRTEM-FFT analysis F1. The needle-like B2-NiAl phases appeared more concentrated in the matrix of alloy 41Z SA compared to other alloys from the SEM-BSE images (Figure 9). The alloy 41Z SA exhibited largest area fraction of needle-like B2-NiAl phases (30%) among the model alloys, while for alloy 43 SA it was reduced to 26%. The fraction of needle-like B2-NiAl phases in the alloys 42+ to 44+ were comparable and was around 20–22% in both as-cast and solution annealed conditions.

Within the needle-like B2-NiAl phases, several bright phases coexisted as encircled in Figure 9. It is apparent that those bright phases are dispersed regularly in solution annealed condition while they are limitedly observed in as-cast condition. As shown in Figure 10, the bright phases dispersed within the B2-NiAl phase are enriched in Fe and Nb based on line scan analysis L2, and correspond to the Laves (Fe_2_Nb) phase from the HRTEM-FFT analysis F2. The Laves phase in the alloys were fine with dimensions ranging from 100–300 nm. In addition, there are light grey colored regions within the dark B2-NiAl phases, which correspond to the Fe- and Cr-enriched sigma phase (σ-phase).

**Figure 11:**
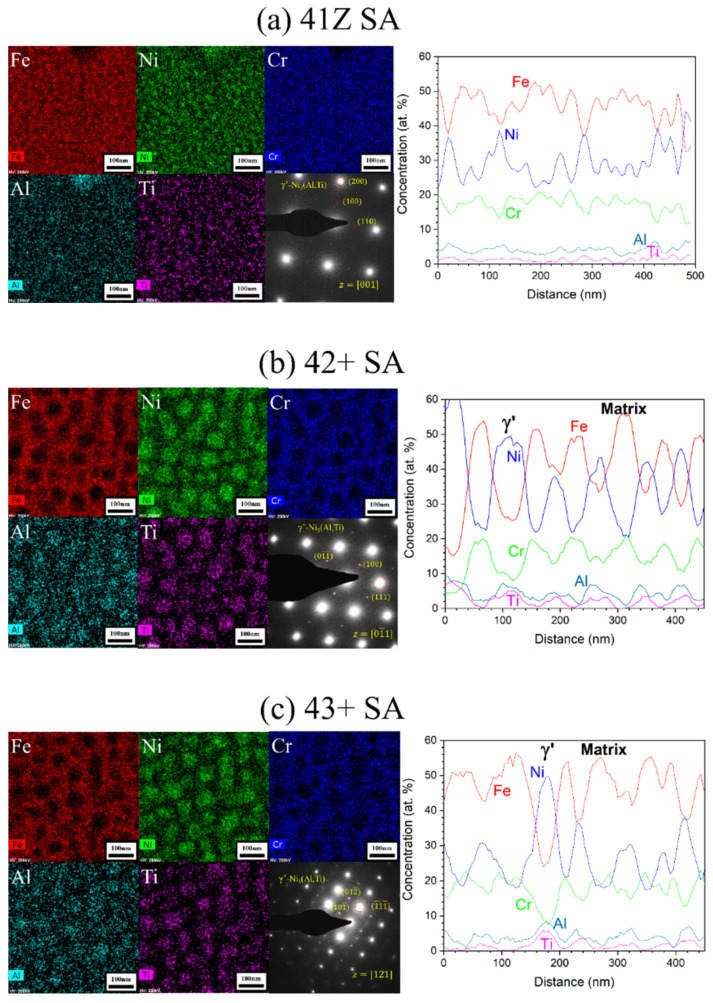
STEM-EDS mapping and line scan analysis results with SAD pattern for the alloys (**a**) 41Z SA, (**b**) 42+ SA, and (**c**) 43+ SA after the creep test in air at 750 °C and 150 MPa.

**Figure 12:**
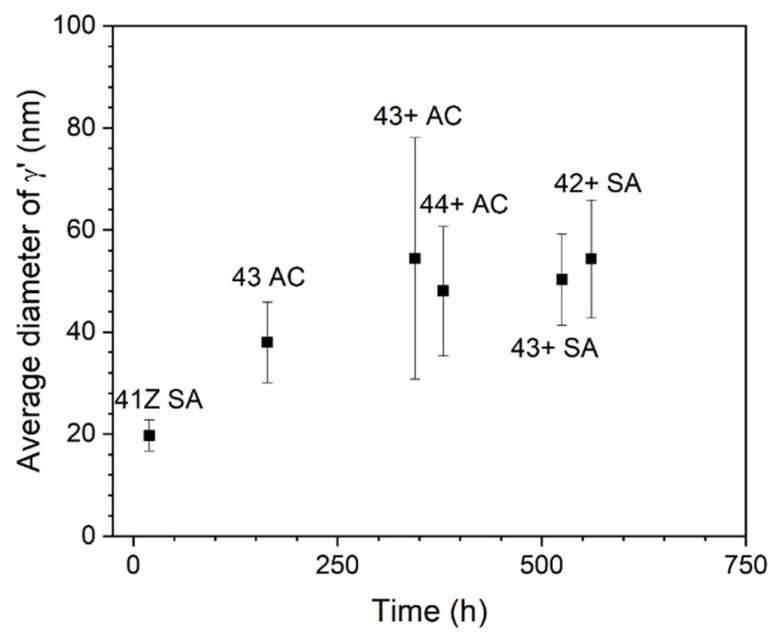
Average diameter of γ’-Ni_3_(Al,Ti) precipitates measured for the creep tested alloys at 750 °C.

The austenite matrix adjacent to the needle-like B2-NiAl phases were further characterized with STEM-EDS analysis and the results are shown in Figure 11. The STEM-EDS mapping (Figure 11a) in the austenite matrix for the crept alloy 41Z SA shows fine cluster-like particles of size less than 20 nm enriched in Ni, Al, and Ti. The SAD pattern contained weaker superlattice points, which corresponds to γ’-Ni_3_(Al,Ti) precipitate. The underdeveloped γ’-Ni_3_(Al,Ti) precipitates observed in the STEM micrograph with weaker SAD pattern suggests the absence of XRD peaks for γ’-Ni_3_(Al,Ti) precipitates in alloy 41Z SA (Figure 8). Meanwhile for the alloys 42+ SA and 43+ SA (Figure 11b,c), the γ’-Ni_3_(Al,Ti) precipitates were clearly developed and exhibited spherical-shaped morphology with the precipitate diameter ranging from 40–70 nm. The SAD pattern contained distinct superlattice points of γ’-Ni_3_(Al,Ti) precipitates with a coherent cube-on-cube orientation relationship with the austenite matrix. The average diameter of the γ’-Ni_3_(Al,Ti) precipitates were measured in some of the alloys exposed to 750 °C for different time periods and are shown in Figure 12. In all cases, the γ’-Ni_3_(Al,Ti) precipitates remained spherical-shaped and coherent with the austenite matrix. Nonetheless, the overall phase fraction of γ’-Ni_3_(Al,Ti) precipitates in the model alloys could not be calculated due to the fine size of precipitates.

**Figure 13:**
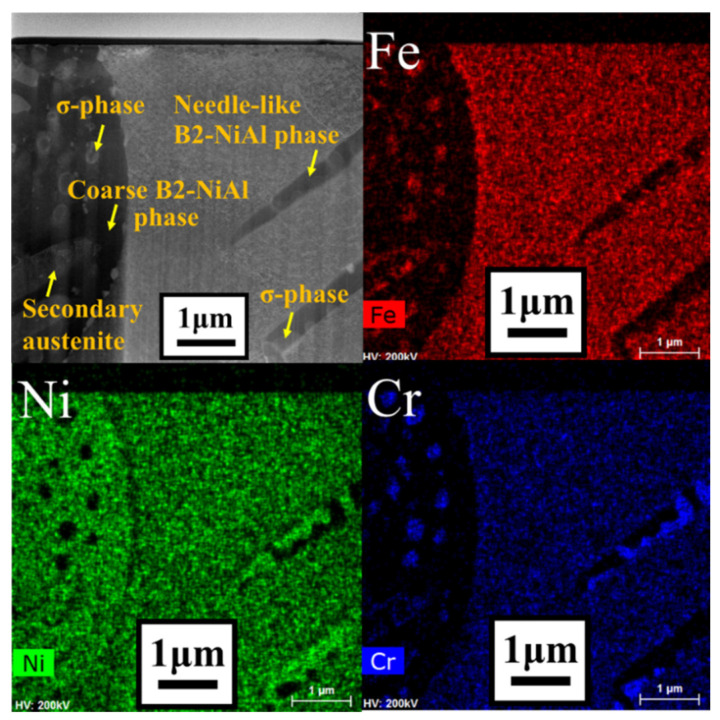
STEM-EDS mapping analysis for the alloy 42+ SA after the creep test in air at 750 °C and 150 MPa.

Apart from the matrix, the coarse B2-NiAl phases also exhibited phase transformation and decomposed into other phases during the creep test at 750 °C. Figure 13 shows the appearance of lighter areas with elongated and globular morphology within the dark, coarse B2-NiAl phases. STEM-EDS mapping in Figure 13 indicated the elongated phases were enriched in Fe and Ni, while the globular phases were enriched in Fe and Cr. Further STEM-EDS line scan analysis with the HRTEM-FFT pattern (not shown here) identified those elongated phases as secondary austenite and globular phases as σ-phase, respectively.

**Figure 14:**
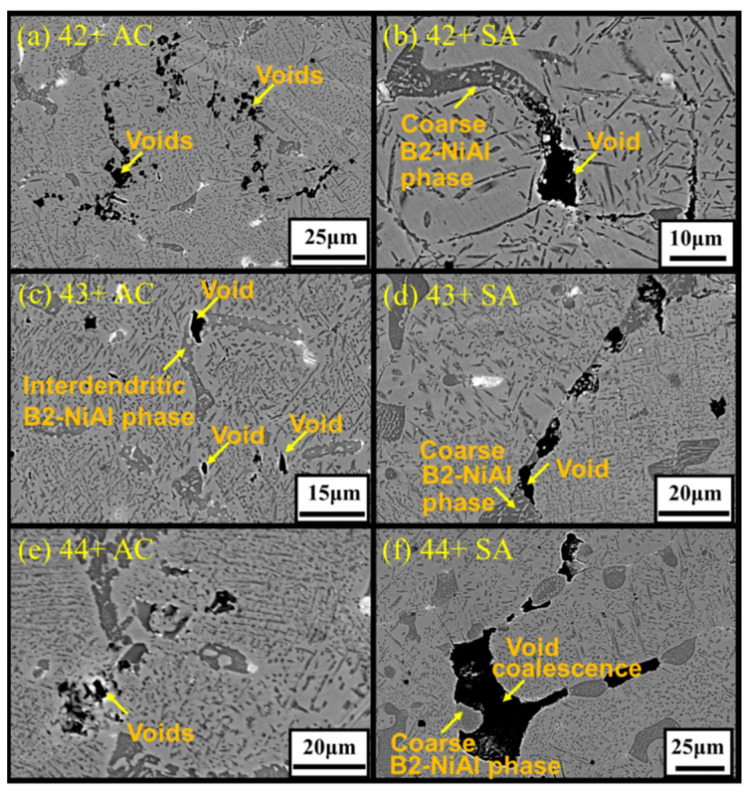
SEM-BSE images showing the microstructure around the fracture region for alloys (**a**) 42+ AC, (**b**) 42+ SA, (**c**) 43+ AC, (**d**) 43+ SA, (**e**) 44+ AC, and (**f**) 44+ SA after the creep test in air at 750 °C and 150 MPa.

**Figure 15:**
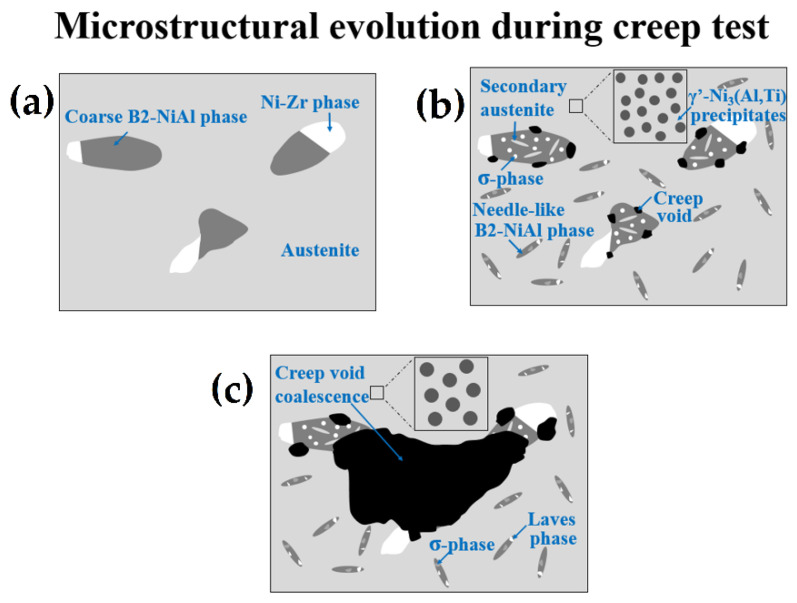
Schematic illustration of the microstructural evolution for the high-Ti alloys (43+ SA and 44+ SA): (**a**) initial microstructure; (**b**) microstructure during creep; (**c**) final microstructure near fracture region.

Figure 14 shows the appearance of creep voids near the fracture region in the crept specimens of model alloys. The creep voids were present in both matrix and within the interdendritic B2-NiAl phases for the as-cast conditions. However, several creep voids nucleated around the coarse B2-NiAl phases in solution annealed conditions, especially for the high-Ti alloys like 43+ SA and 44+ SA. Furthermore, the coalescence of the neighboring creep voids resulted in crack propagation along the coarse B2-NiAl phases, especially for the alloys 43+ SA and 44+ SA with more and larger coarse B2-NiAl phases as shown in Figure 14. The overall microstructural evolution during the creep test for the high-Ti alloys (43+ SA and 44+ SA) is illustrated in Figure 15.

## 4. Discussion

The addition of Ti in alumina-forming alloy is generally limited due to the consideration of high-temperature oxidation properties. Yamamoto et al. [11] added 2 wt% Ti for the 32Ni–3Al alloy, which offered considerable increase in creep rupture life and oxidation resistance in presence of beneficial minor elements Zr, C and B. However, the relatively low 3 wt% Al content would be a concern for long-term high-temperature oxidation resistance due to the gradual subsurface Al depletion and additional presence of Ti. Enough Al content is necessary to avoid deterioration of the oxidation resistance and, therefore, heat-resistant alloys with 4.5 wt% Al and γ’-Ni_3_(Al,Ti) precipitates are explored in this study. The higher Al content in the model alloys necessitate higher Ni content for the formation of γ’-Ni_3_(Al,Ti) precipitates instead of B2-NiAl phases. Meanwhile, Ti also encourages the formation of the γ’-Ni_3_(Al,Ti) precipitates and enhances its thermodynamic stability. Increasing Ti is a concern for the high-temperature oxidation resistance, while the higher Al content with beneficial minor elements (Zr, C and B) would lessen the detrimental effects of Ti. The high-temperature oxidation tests are currently being studied in S-CO_2_ and steam environments, and will be reported separately.

The higher Al and Ti content in the model alloys caused micro-segregation of Al and Ti into the B2-NiAl interdendritic phases upon solidification. The solution annealing at 1200 °C partially dissolved the B2-NiAl interdendritic phases in which the coarsened B2-NiAl phases were still observed (Figure 2). The presence of coarse B2-NiAl phases after solution annealing at 1200 °C was not expected as the predicted solvus temperatures was around 1100 °C (Figure 1b). To completely dissolve the B2-NiAl phases, longer annealing time may be needed. Nevertheless, the fraction of the coarse B2-NiAl phases increases with Ti (Figure 4) as indicated from the phase stability calculations in Figure 1b, attributed to the limited solubility of Ti in the austenite matrix. In addition, the coarse B2-NiAl phases were unstable during creep testing at 750 °C, which decomposed into secondary austenite and σ-phase (Figure 15).

Moreover, the needle-like B2-NiAl phases appeared considerably after the creep test at 750 °C (Figure 9), though the alloys 43+ and 44+ had limited stability of B2-NiAl phases around 750 °C based on the phase stability calculation (Figure 1b). Additionally, the needle-like B2-NiAl phases were also present beneath the oxidation-tested specimens (not shown here), indicating that it is more associated with their higher thermodynamic stability rather than the stress during creep test. The increased Ni content from alloy 30Ni–1Ti (41Z) towards 35Ni–(2~4)Ti alloys (43, 42+, 43+, and 44+) effectively reduced the fraction of needle-like B2-NiAl phases during the creep test (Figure 9). Meanwhile, increasing the matrix Ti content for the alloys 42+ to 44+ did not noticeably altered the fraction of needle-like B2-NiAl phases in both as-cast and solution annealed conditions. The alloy 43+ contained slightly lower fraction of needle-like B2-NiAl phases than its counterpart alloy 43, which could be attributed to the presence of Zr that was reported to reduce the B2-NiAl phase fraction [11]. The alloy 41Z exhibited the least creep rupture life indicating that those extensively precipitated needle-like B2-NiAl phases are inadequate for improving the creep rupture life at 750 °C. Nevertheless, the B2-NiAl phases are considered beneficial as they may act as Al-reservoirs to form a protective alumina layer during high-temperature oxidation [26]. Furthermore, the coexistence of fine Laves and σ-phases within the needle-like B2-NiAl phases can be explained by the rejection of elements like Fe, Cr and Nb during the growth of needle-like B2-NiAl phases. The segregated elements then involve in the nucleation of their respective phases.

The γ’-Ni_3_(Al,Ti) precipitates in the alloy 41Z SA were underdeveloped with the appearance of cluster-like particles, while clearly developed γ’-Ni_3_(Al,Ti) precipitates were observed in alloys 42+ SA and 43+ SA (Figure 11). This can be attributed to the shorter exposure time at 750 °C for the alloy 41Z SA corresponding to shorter creep rupture life. The increased creep rupture life from alloy 30Ni–1Ti (41Z) towards 35Ni–(2~4)Ti alloys (43, 42+, 43+ and 44+) can be attributed to the increased Ni and Ti content. The extensive B2-NiAl precipitation in alloy 41Z SA suggests that γ’-Ni_3_(Al,Ti) precipitates were insufficient to provide longer creep rupture life. Furthermore, the increase in Ti content (alloys 42+ to 44+) increased the tensile strength and creep rupture life in as-cast condition, as described in Section 3.3. It seems that the increased Ti content would have enhanced the γ’-Ni_3_(Al,Ti) precipitate fraction in the austenite matrix as observed from the phase stability calculation in Figure 1a, that contributed to the increased creep rupture life. However, the overall fraction of γ’-Ni_3_(Al,Ti) precipitates in the model alloys could not be accurately estimated because of its fine size. Additionally, the solution annealing effectively increased the matrix Ti content and resulted in improvement in the creep rupture life (Figure 5 and Figure 7). In addition, the increased matrix Nb content after solution annealing stabilized the fine Laves phases (Figure 9), which provides additional creep resistance [19]. Nevertheless, no direct correlation between Ti content and creep rupture life could be obtained for 35Ni–(2~4)Ti alloys in the solution annealed condition (Figure 7). The considerable creep void nucleation and crack propagation along the coarse B2-NiAl phases for high-Ti alloys (Figure 14) could explain such observation. For high-Ti alloys, the increase in fraction and size of coarse B2-NiAl phases after solution annealing resulted in a considerable increase of creep void formation and their coalescences during creep test. Therefore, the high-Ti alloys 43+ SA and 44+ SA could have failed somewhat earlier than expected, and the beneficial effect of higher Ti content was not fully observed. Therefore, it is important to reduce or eliminate the coarse B2-NiAl phases to achieve the full benefit of increased Ti content in the matrix.

## 5. Conclusions

The cast Fe-Ni-based heat-resistant alloys with 4.5 wt% Al and varying Ti content were studied for the effect of Ti content on high-temperature creep rupture life and microstructural evolution.

1. The as-cast alloys showed dendritic microstructure with B2-NiAl and Ni-Zr rich interdendritic phases. Solution annealing at 1200 °C partially dissolved the interdendritic phases while some coarse B2-NiAl and Ni-Zr rich phases existing. The fraction and size of the coarse B2-NiAl phases after solution annealing increased with Ti content. Solution annealing also increased the Ti and Nb content in the austenite matrix.
2. The 35Ni–(2~4)Ti alloys showed much increased creep rupture life compared to 30Ni–1Ti alloy, attributed to the increase in γ’-Ni_3_(Al,Ti) precipitates from higher Ni and Ti content. Among the 35Ni–(2~4)Ti alloys, increasing Ti content from 2 to 4 wt% gradually increased the creep rupture life in the as-cast condition.
3. The model alloys consisted of nano-sized, spherical-shaped, and coherent γ’-Ni_3_(Al,Ti) precipitates that enhanced the creep rupture life. Extensive precipitation of needle-like B2-NiAl phases was observed but inadequate for improving the creep rupture life at 750 °C. Fine Laves phase and σ-phase were present adjacent to the needle-like B2-NiAl phases. The Laves phase was dispersed regularly in solution annealed condition while it was limitedly observed in the as-cast condition.
4. Solution annealing improved the creep rupture life of the alloys compared to as-cast condition, attributed to the increased Ti and Nb content in austenite matrix. Nevertheless, no direct correlation between Ti content and creep rupture life could be obtained for 35Ni–(2~4)Ti alloys in the solution annealed condition. For high-Ti alloys, the increase in fraction and size of coarse B2-NiAl phases after solution annealing resulted in considerable increase of creep void formation and their coalescences during creep test, and the beneficial effect of higher Ti content was not fully achieved.

## Figures and Tables

**Table 1:** Chemical composition in wt% for the model heat resistant alloys.

Alloys	Fe	Ni	Cr	Al	Ti	Nb	Si	Zr	C	B
41Z	Bal.	30	18	4.5	1	1	0.2	0.3	0.025	-
43	Bal.	35	18	4.5	3	1	-	-	0.025	-
42+	Bal.	35	16	4.5	2	1	0.2	0.3	0.08	0.015
43+	Bal.	35	16	4.5	3	1	0.2	0.3	0.08	0.015
44+	Bal.	35	16	4.5	4	1	0.2	0.3	0.08	0.015

**Table 2:** Average composition in at% from SEM-EDS analysis for interdendritic phases in alloy 43+ in as-cast (AC) and solution annealed (SA) conditions.

Alloy	Phase	Fe	Ni	Cr	Al	Ti	Nb	Si	Zr
43+ AC	B2-NiAl phase	13.2	42.4	4.6	23.4	12.9	1.4	0.9	1.3
	Ni-Zr phase	15.2	42.3	5.2	8.0	2.7	3.2	11.7	11.7
43+ SA	B2-NiAl phase	22.3	39.8	8.1	21.9	6.9	0.4	0.4	0.2
	Ni-Zr phase	18.5	49.5	5.2	5.2	5.0	2.4	2.9	11.3

**Table 3:** Tensile test results of the model alloys in as-cast condition at 700 and 750 °C.

Alloy	700 °C Tensile Test			750 °C Tensile Test		
	Yield Strength, MPa	Tensile Strength, MPa	Elongation, %	Yield Strength, MPa	Tensile Strength, MPa	Elongation, %
41Z	331.1	493.5	41.8	309.6	385.2	30.3
43	514.7	593.2	11.0	503.3	553.7	18.7
42+	509.9	614.7	33.1	479.1	511.8	42.1
43+	612.3	711.9	17.3	492.6	553.5	35.9
44+	665.6	737.4	17.3	521.7	551.9	43.3

## Data Availability

The data presented in this study are available on request to the corresponding author.
